# Employing human rights frameworks to realize access to an HIV cure

**DOI:** 10.7448/IAS.18.1.20305

**Published:** 2015-11-13

**Authors:** Benjamin Mason Meier, Adriane Gelpi, Matthew M Kavanagh, Lisa Forman, Joseph J Amon

**Affiliations:** 1 Department of Public Policy, University of North Carolina, Chapel Hill, NC, USA; 2 Edmond J. Safra Center for Ethics, Harvard University, Cambridge, MA, USA; 3 Department of Political Science, Leonard Davis Institute of Health Economics, University of Pennsylvania, Philadelphia, PA, USA; 4 Dalla Lana School of Public Health, University of Toronto, Toronto, Canada; 5 Health and Human Rights Division, Human Rights Watch, New York, NY, USA

**Keywords:** HIV cure, human rights, right to health, operations research, global governance, essential medicines

## Abstract

**Introduction:**

The scale of the HIV pandemic – and the stigma, discrimination and violence that surrounded its sudden emergence – catalyzed a public health response that expanded human rights in principle and practice. In the absence of effective treatment, human rights activists initially sought to protect individuals at high risk of HIV infection. With advances in antiretroviral therapy, activists expanded their efforts under international law, advocating under the human right to health for individual access to treatment.

**Discussion:**

As a clinical cure comes within reach, human rights obligations will continue to play a key role in political and programmatic decision-making. Building upon the evolving development and implementation of the human right to health in the global response to HIV, we outline a human rights research agenda to prepare for HIV cure access, investigating the role of human rights law in framing 1) resource allocation, 2) international obligations, 3) intellectual property and 4) freedom from coercion.

**Conclusions:**

The right to health is widely recognized as central to governmental, intergovernmental and non-governmental responses to the pandemic and critical both to addressing vulnerability to infection and to ensuring universal access to HIV prevention, treatment, care and support. While the advent of an HIV cure will raise new obligations for policymakers in implementing the right to health, the resolution of past debates surrounding HIV prevention and treatment may inform claims for universal access.

Human rights have been central to the global response to the HIV pandemic, providing a legal foundation for social, political and institutional efforts to protect vulnerable individuals and fulfil government obligations. As medical advances bring the world closer to a clinical cure, new rights-related issues are emerging. Even as scholars address the ongoing ethics and rights challenges associated with clinical trials to develop such a cure, operations research is needed now on the future human rights considerations associated with realizing access to a prospective HIV cure.

Diverse efforts to develop an HIV cure are currently being undertaken, with varied implications for both public health interventions and human rights obligations. Studies are underway to discover methods of both viral eradication and sustained virologic remission through radioimmunotherapy (to kill HIV-infected cells), stem cell transplantation (to make cells impervious to reinfection) and antiretroviral (ARV) therapy (to achieve long-term sustained viral suppression). An eventual cure may result from one of these efforts or some combinations in tandem. Yet even as a clinical cure remains remote – with only one individual to date considered to have been “clinically cured” of HIV – the broad contours of a human rights response to cure access can and should be considered to anticipate and guide human rights concerns that may arise.

This article examines the potential role of international human rights obligations in the future global response to HIV. Tracing the evolution of human rights in the HIV pandemic, the introduction chronicles the paths through which medical advances have influenced human rights claims and human rights claims have framed the global response to prevention and treatment. Given scientific hope that a clinical cure may soon exist, our discussion analyzes the ways in which human rights law can frame access to an HIV cure, developing these obligations under the human right to health and implementing these obligations to overcome barriers to access. Concluding that human rights can provide a means to frame state obligations, we outline a human rights research agenda to prepare for access to an HIV cure.

## Introduction: the evolving role of human rights in responding to the HIV pandemic

The initial human rights response to the emerging HIV epidemic was local. In the United States, Europe and Australia, sex workers (SW), men who have sex with men (MSM) and injection drug users (IDU), concerned about their vulnerability and that of their peers, created new organizations for outreach and education. These organizations – working with politically marginalized, and often criminalized, populations and sometimes providing illegal services (e.g. needle and syringe exchange) – fought to end government inaction and lack of funding (both national and local) and to ensure government protection from stigma, discrimination and violence [1]. Beyond a focus on individual behaviours, these organizations began to understand human rights abuses as structural determinants of health, suggesting a “political epidemiology” linking the impact of laws, policies and their enforcement with health outcomes [2, 3].

Ignorance and fear of a new and deadly disease in the early years of the epidemic led governments to violate rights to privacy as a presumptive (yet wholly inadequate) means of preventing transmission. Mandatory HIV testing and disclosure was demanded in a wide variety of settings, including among healthcare providers and patients, employees and employers, school children and officials, and intimate partners [4]. Recognizing the ways in which these human rights violations created vulnerability to HIV infection, rights activists and public interest lawyers advocated for the integration of human rights principles into public health policy, viewing discriminatory public health programmes as counterproductive to public health goals and applying human rights protections to focus on the individual risk behaviours leading to HIV transmission [5]. Even as many nations continued to violate human rights in the HIV response (with many violations continuing into the present), nations such as Uganda, Thailand and Brazil were driven by activist pressures in the mid-1980s to acknowledge their obligations to respond to the epidemic by adopting pragmatic programmes that openly and frankly addressed HIV, fostering civil society participation and engaging affected communities to help design, implement and evaluate programmes to prevent HIV transmission and care for the sick [6]. The World Health Organization (WHO) Global Programme on AIDS sought in the late 1980s to extend human rights efforts globally, promoting recognition of the “inextricable linkages” between public health and human rights in the global HIV response and developing a rights-based framework for global health governance and national AIDS prevention plans [7–9].

Transforming national and global governance, the promise of medical treatment became a reality with the 1987 approval of zidovudine, the first ARV to treat HIV. However, resource constraints tempered hopes for treatment access, with international funding limitations (combined with arguments about the feasibility of HIV treatment in low-resource settings) serving as a continued barrier to treatment through the 1996 introduction of combination therapy [10]. As infection rates continued to climb in developing countries and life-saving therapy remained inaccessible to the overwhelming majority of those living with HIV, human rights activists employed the right to health to demand access to treatment under international law.

The UN Committee on Economic, Social and Cultural Rights (CESCR) took up these evolving legal issues in its 2000 General Comment on the human right to health. With a UN mandate to interpret the International Covenant on Economic, Social and Cultural Rights, the CESCR interpreted obligations for “disease prevention, treatment and control” to include specific state responsibilities for “the provision of essential drugs” and “the establishment of prevention and education programmes for behaviour-related health concerns such as sexually transmitted diseases, in particular HIV/AIDS …” [11]. The CESCR's application of human rights to the pandemic, in ways similar to other UN treaty bodies’ consideration of the rights of women and children in the context of HIV, sought to de-emphasize individual treatment while recognizing the influence of public health prevention in addressing the interconnected population-level determinants of HIV transmission [12, 13]. At the intersection of individual and public health, the CESCR examined disease prevention and health promotion efforts to progressively realize the right to health, providing immediate and resource-dependent obligations to ensure the availability, accessibility, acceptability and quality of health systems and services [11].

Reflecting this emerging consensus on human rights to prevention and treatment in global governance, an historic 2001 United Nations General Assembly Special Session (UNGASS) on HIV/AIDS viewed the pandemic not only as a human rights challenge and a public health crisis, but also as a threat to international security [14]. The UNGASS put virtually all of the world's leaders on record as endorsing a specific set of global targets for combating HIV, with its formal declaration explicitly acknowledging the links between the spread of HIV and poverty, underdevelopment and illiteracy [15]. While recognizing that structural determinants of ill health – including stigma, discrimination, lack of confidentiality and gender inequality – undermined prevention and care efforts, the declaration also affirmed that access to medicine was fundamental to the realization of the right to health [16].

The recognition of a right to medicines was also advanced by civil society through litigation, seeking to hold states accountable under the human right to health for individual access to treatment [17, 18]. Building on the success of previous judicial claims in Latin America and Southeast Asia, the South African Constitutional Court heard a rights-based challenge for access to medicines in the seminal 2002 case *Minister of Health v. Treatment Action Campaign* [19]. Brought pursuant to South Africa's constitutional codification of a right to access healthcare, this legal challenge successfully held the government responsible for expanding drug access to reduce the transmission of HIV from mother to child [20]. Emphasizing legal accountability for the human right to health, this evolving trend towards litigation throughout the world illustrates that the South African case is not exceptional and that successful human rights litigation is occurring in countries such as Colombia, Brazil, Costa Rica, Kenya and India, as litigants seek to assure that essential medicines are accessible to all [21–23].

As activists attacked global inequality in access to HIV treatment as a matter of social justice, international funding debates became central to human rights considerations under international law. The CESCR returned to the right to health in its 2006 General Comment, finding that states “have a duty to prevent unreasonably high costs for access to essential medicines … from undermining the rights of large segments of the population to health” [24]. Recognizing the financial limitations of developing states in providing affordable medications, civil society advocates soon broadened their right to health advocacy (through public demonstration, government lobbying and legal action) to implicate international obligations on all manner of powerful states, organizations and corporations with the ability either to support or to impede access to ARVs in the developing world [25, 26]. Moved by the scale of the pandemic, wealthy nations came together to coordinate their financial allocations to secure “universal access,” mobilizing unprecedented resources for global health [27]. The UN Special Rapporteur on the right to health took up the global challenge of securing access to medicines, finding in 2006 that the “human right to medicines” is an “indispensable part” of the right to health and holding that “states have to do all they reasonably can to make sure that existing medicines are available in sufficient quantities”[28].

These human rights obligations have been increasingly recognized in national government and international organization strategies for HIV prevention, treatment, care and support. The Joint United Nations Programme on HIV/AIDS (UNAIDS), the Global Fund against HIV, TB and Malaria (Global Fund), and the US President's Emergency Plan for AIDS Relief (PEPFAR) all now recognize the protection and fulfilment of human rights as a core strategy in their response. This emphasis on international human rights obligations has come at the same time that increasing attention is being paid to a more comprehensive response to HIV [29] and an ongoing need to address political determinants of HIV vulnerability [2]. Recalibrating human rights law to reject trade-offs between treatment and prevention, programmatic guidelines and budgetary allocations have come to focus on rights-based universal access, emphasizing a comprehensive approach to prevention, treatment, care and support in global health governance [30]. This mutually reinforcing human rights approach to prevention, treatment, care and support has been buttressed scientifically, as epidemiologic evidence has shown that viral suppression through ARVs could prevent HIV transmission, linking individual treatment to collective prevention [31]. With scientific consensus emerging that ARV treatment should be started immediately upon diagnosis, improving individual outcomes and protecting uninfected populations, proponents have stressed both the rights-based complementarity of “treatment as prevention” and the importance of maintaining attention to human rights protections [32–34].

As rights-based governance has evolved to progressively realize rights of access to prevention, treatment, care and support, it is necessary to consider the next step in this individual and collective human rights analysis: providing access to a prospective HIV cure.

## Discussion: human rights and access to an HIV cure

With over 35 million estimated to be living with HIV, the development of a cure for HIV would be a triumph for medicine and an opportunity for public health. HIV cure research is drawing on modalities from HIV prevention and treatment research to investigate the infection and disease progression spectrum. A variety of modalities for an effective cure are being explored, including sterilizing interventions that would purge the viral reservoir and actions that would push the virus into remission sufficiently to allow viral suppression without the need for ARV medications [35, 36]. In analyzing the prospective clinical manifestations, the human rights implications will depend on whether the cure is:

1. Universally effective, where an HIV cure could be effective for the vast majority of HIV-positive individuals as a one-time (or short course) intervention and provide long-lasting immunity from reinfection or
2. Partially effective, where an HIV cure could be effective for only some of the population, could eradicate only part of the viral load, could require repeated boosters for full immunity or could provide only limited periods of protection from future reinfection [37, 38].

Whereas a universally effective cure that provides long-lasting immunity would provide both treatment and prevention benefits, a progressively rolled-out, partially effective cure could potentially raise human rights conflicts in some countries in prioritizing resources for access to treatment, prevention and cure.

Ensuring that an HIV cure is able to fundamentally alter the trajectory of the HIV pandemic requires human rights analysis in two key areas: framing government obligations under the right to health and addressing barriers to universal access to a cure under a rights-based approach to health.

### Government obligations to provide universal access to an HIV cure

The obligations of governments to provide access to an HIV cure would, in principle, be little different than the established recognition of government obligations to ensure access to HIV treatment. Government obligations under the human right to health, grounded in the evolution of international human rights treaty law, guarantee the right of everyone to the highest attainable standard of health and require states to take steps to realize this right through core obligations to realize access to essential medicines [11]. In realizing this right, it is highly likely that an HIV cure, similar to ARVs, would be classified by the WHO as an essential medicine, raising core national and international obligations to provide access immediately without regard to resources. Beyond this core obligation, states would bear additional obligations to progressively realize the right to health. This state obligation to progressively realize the right to health demands that national resources and international assistance be committed to the government's “specific and continuing obligation to move as expeditiously and effectively as possible towards the full realization of [the right]” [11, 39].

The human right to health therefore provides a legal framework for developing obligations to prioritize access to an HIV cure based upon its *availability*, *accessibility*, *acceptability* and *quality*.

1. *Availability* requires that a cure be provided in sufficient quantities for the affected population, including sufficient quantities of essential medicines, health personnel and other mechanisms for distribution [11]. At the global level, it has often taken five or more years for new HIV treatments to become widely available in the developing world due to regulatory, logistic and cost hurdles. With evidence that there is no “safe” period of time to live with unsuppressed HIV, a rights-based approach would seek advanced planning to assure rapid global distribution of an HIV cure, establishing new regulations to assure that any commercialized research will receive simultaneous registration and begin immediate production for global availability.
2. *Accessibility* of a cure looks to physical accessibility (providing the cure within safe, physical reach of all, especially marginalized populations), information accessibility (providing information about a cure to affected populations while respecting patient confidentiality) and financial accessibility (ensuring that the cure is affordable for all) [11]. Different cure modalities will require different types of health systems, workforce, pricing and infrastructure to ensure that the cure reaches those who need it, each presenting equity concerns for those without current access to health systems due to social, economic or geographic marginalization. Especially for a partially effective cure, the obligation to ensure access to treatment will remain a human rights imperative for those who cannot access a cure or for whom a cure is not effective.
3. *Acceptability* of a cure requires that interventions account for differences across populations (e.g. gender, race, culture, sexual orientation) while respecting medical ethics and informed consent [11]. Choice is of paramount importance for any medical intervention, and it will be necessary to consider the lived reality of access, considering issues of vulnerability and marginalization that could make the cure unacceptable [40–42]. There may be significant side effects of a cure or significant burdens placed on those undertaking a cure (travel, lost work, financial cost, particular biological requirements). As a result, it is likely that states will need to provide a cure opportunity to all while avoiding the real or implied punishment of removal from ARVs to those for whom a cure does not work or who do not choose curative interventions [43].
4. *Quality* requires that an HIV cure be “scientifically and medically appropriate and of good quality,” including cure provision by skilled medical personnel and distribution mechanisms to improve the health of affected patients [11]. The quality of any prospective cure will necessitate global standards to ensure clinical effectiveness and alleviate side effects.

This availability/accessibility/acceptability/quality matrix under the human right to health provides a framework through which to evaluate efforts to implement obligations for HIV cure access.

To assure that the realization of access to an HIV cure is universal, international law also establishes overarching principles of non-discrimination and equality. The Universal Declaration of Human Rights proclaims that

[e]veryone is entitled to all the rights and freedoms set forth in this Declaration, without distinction of any kind, such as race, colour, sex, language, religion, political or other opinion, national or social origin, property, birth or other status.

In facilitating non-discriminatory access to an HIV cure as a core component of the right to health, fundamental equity concerns will arise concerning disparities in testing, treatment and cure across the population, including to vulnerable and at-risk populations such as women, children, MSM, IDUs and SWs [44]. States must address the current evidence of barriers for these vulnerable and at-risk populations and take pro-active steps to overcome these barriers from the start of HIV cure programmes.

### Barriers to access to an HIV cure

Looking beyond the obligations to realize universal access to an HIV cure, it is important to consider the obligations of states to implement programmes to address the human rights barriers that may emerge. Given the evolving ways that the right to health has been mobilized in HIV policy, there are lessons that can be applied in framing HIV cure access. To operationalize these principles in public policy, building upon the rights-based mechanisms that evolved through HIV prevention and treatment, the implementation of the right to health in an HIV cure could have interconnected impacts on 1) resource allocation, 2) international obligations, 3) intellectual property and 4) freedom from coercion.

#### Resource allocation

Where the progressive realization of the right to health requires states to expend the maximum available resources, a cure could provide a new set of rights-based obligations for an expansion of resources for access and a more efficient allocation of resources towards a cure. An HIV cure, much like HIV treatment before it, could come to fall within the core minimum obligations of states under the right to health, conceptualized as an “essential medicine” and/or a “measure to prevent, treat and control epidemic disease” [11]. Even as a cure entails benefits to both infected individuals and uninfected populations, the progressive realization of access to a cure will require rights-based policy to adopt a two-pronged approach: continuing to ensure the protection of vulnerable uninfected populations through HIV prevention while also transitioning those individuals on HIV treatment towards a cure. In reallocating resources across prevention, treatment and cure, the right to health would not support the premature dismantling of prevention and treatment programmes whose maintenance and expansion will be critical for the realization of human rights, especially where the cure is either partially effective or prohibitively expensive. While inequalities in access to a cure will make continued support for treatment vital, human rights would support those currently on treatment being shifted voluntarily to a cure as it becomes available, progressively freeing resources currently devoted towards treatment [45]. Learning from the ways in which past cures have been seen to diminish the political will for prevention [46, 47], a health system must continue to provide meaningful opportunities to prevent HIV infection (or reinfection) to protect the collective rights of populations without HIV [48].

#### International obligations

In this effort to implement access for a cure, human rights related to an HIV cure implicate not only the most affected developing states, but all states with the ability to support or impede access to a cure. The right to health bears obligations of “international assistance and cooperation,” looking to the international obligations of wealthy countries to “facilitate access to essential health facilities, goods, and services in other countries, whenever possible, and provide the necessary aid when required” [11]. Extended to global governance, including those international institutions that assist and cooperate with states in the realization of human rights, this human rights framework suggests that, at minimum, international organizations would take on explicit new tasks to convene, coordinate and promote rights-based cure access [49]. As with prevention and treatment access, an advance global agreement may be needed to ensure this assistance and cooperation, both to fund the cure itself (in single or multiple-doses) and to build out the health systems necessary for a successful rights-based rollout of an HIV cure (building on existing prevention networks and treatment delivery systems, especially for a partially effective cure that will require regular follow-up diagnostics, boosters and clinical visits).

#### Intellectual property

These international obligations will depend crucially on the flexibility of the international intellectual property system, through which exorbitant costs for a patented cure may deny access to all but a privileged few. Intellectual property protections will create challenges to affordability, as seen initially with the costs of HIV treatment and now with the introduction of a hepatitis C virus cure [50–52]. Yet although the World Trade Organization (WTO) Agreement on Trade-Related Intellectual Property Rights (TRIPS) requires national recognition of patents on medicines, WTO members may adopt measures necessary to protect public health through parallel importation (importing cheaper patented medicines), compulsory licensing (to manufacture or import generics), high standards of patentability, exceptions for least developed countries and other measures. Subsequent WTO agreements confirm that TRIPS “does not and should not prevent members from taking measures to protect public health” and that TRIPS “should be interpreted and implemented in a manner supportive of a WTO member's right to protect public health and, in particular, to promote access to medicines for all.” Nevertheless, states are likely to face opposition in making use of these legal flexibilities to lower the costs of purchasing a prospective cure [53, 54]. Given how international trade laws and practices have constrained access to other essential medicines in the context of public health emergencies, the challenge of balancing the right to health with intellectual property rights is likely to persist as an HIV cure becomes a reality.

#### Freedom from coercion

The rollout of a cure will require governments to take pro-active steps to reduce the marginalization that underlies stigma, discrimination and violence. Currently, more than 50% of new HIV infections occur among five key populations (MSMs, SWs, individuals in prisons or other closed settings, transgender people, and IDUs) that are frequently marginalized and criminalized [55]. The development of policies and programmes to facilitate access to an HIV cure must include participation from at-risk populations and the realization of calls – by UNAIDS, the WHO and others – to decriminalize homosexual sex, sex work and individual drug use and possession [56, 57]. Increasing access to a cure for populations in closed settings may require both improved healthcare in these settings and criminal justice reform [58]. Although principles of informed consent, individual counselling and patient confidentiality are central to established HIV guidelines, concerns must be addressed that an HIV cure will resurrect debates on widespread mandatory HIV testing and subject people living with HIV to stigma, discrimination and violence [59]. Learning from the unintended consequences of previous cures, individuals who do not seek an HIV cure – whether because of medical contraindications, lack of access or personal choice – should be protected from forced treatment, coercion, criminal penalties, discrimination and stigma [60].

## Conclusions: structuring a human rights research agenda on HIV cure access

As a cure comes within reach, the right to health can continue to provide a normative framework for shaping the global HIV response. The development of a clinical cure will reconceptualize the “highest attainable standard” of health under international human rights law, likely creating immediate and resource-dependent obligations for national governments, international organizations and civil society to realize access to an HIV cure as an “essential medicine.” Yet vaccines and cures for other widespread diseases already exist, with dishearteningly little effect on access to these clinical advancements. Given the evolution of the right to health in addressing HIV, frameworks for progressively realizing access to a cure can build upon past work for access to prevention, treatment and care. These frameworks must consider how human rights might differentially affect access to a cure where the realities of inadequate access would likely mean a mix of those cured of HIV, those continuing to live with HIV and those at high risk of HIV infection.

Through an expanded operations research agenda on human rights, policymakers can prepare for this next great challenge by addressing the following issues:

1. Allocating resources: In prioritizing access for a cure, considering equity in implementing the principle of progressive realization, it will be necessary to consider the rights-based allocation of resources across access to cure, treatment (especially where the cure does not have perfect efficacy) and prevention (especially where the cure does not confer future immunity), including the rights-based programmatic guidance necessary to account for a new cure regimen.
2. International obligations: While global health governance is becoming increasingly sensitive to human rights issues [61], the implications of a cure have not yet been sufficiently analyzed, and the WHO (as the normative/technical agency), UNAIDS (as the joint political programme) and the Global Fund (as the financing body) must consider their human rights obligations to facilitate cure access in partnership with each other [62, 63].
3. Intellectual property: Given tiered pricing strategies pursued by multinational pharmaceutical companies, which can still leave drugs unaffordable for many in states with large HIV burdens, scholars can investigate open licensing strategies for cure research, government strategies to use TRIPS flexibilities to import or manufacture cheaper medicines and civil society strategies to lower prices and facilitate the availability of generics [54].
4. Freedom from coercion: As with other empirical predictions of the potential health consequences of an HIV cure, such predictions must be balanced against potential increases in stigma, discrimination and violence, learning from the continuing marginalization attached to other treatable sexually transmitted infections [64] to understand how the development of a cure may affect HIV testing approaches and raise fears of increased high-risk behaviours [65–68].

The development of a cure for HIV will likely raise some of the same human rights dilemmas that past medical advancements provoked around resource allocations, international obligations, intellectual property and freedom from coercion. Yet, while an HIV cure will likely resemble past initiatives, the prospect of a cure for HIV also raises distinct challenges at the intersection of health and human rights, creating an imperative for human rights scholars and advocates to prepare for the challenges to come.
